# Case report: Pyrotinib in the treatment of advanced scrotal EMPD combined with sweat gland carcinoma

**DOI:** 10.3389/fonc.2024.1382376

**Published:** 2024-07-09

**Authors:** Liquan Zhu, Haoqiang Wang, Donghai Cheng, Wei Wang, Yue Lei, Ning Yang, Lijun Peng, Peng Liu, Juan Zhou, Bo Xie

**Affiliations:** ^1^ Department of Oncology, General Hospital of Southern Theater Command, Guangzhou, China; ^2^ The First School of Clinical Medicine, Southern Medical University, Guangzhou, China; ^3^ Department of Pathology, General Hospital of Southern Theater Command, Guangzhou, China; ^4^ Graduate School, Guangzhou University of Traditional Chinese Medicine, Guangzhou, China

**Keywords:** pyrotinib, extramammary Paget disease (EMPD), sweat gland carcinoma, targeted therapy, human epidermal growth factor receptor 2

## Abstract

Extramammary Paget disease (EMPD) is a rare cutaneous intraepithelial adenocarcinoma, which is mostly distributed in areas with sweat gland cells and mainly occurs in the anogenital skin of women. The male genital tract involvement is extremely rare and often occurs with other malignant tumors. Paget’s disease in the scrotum with sweat gland carcinoma is even rarer. In the first report of scrotal endocrine sweat gland carcinoma associated with Paget disease by Saidi et al. in 1997, no more than 50 cases have been reported in the relevant research worldwide. Early EMPD combined with sweat gland carcinoma is mainly surgical treatment, and there is no standard treatment plan for advanced EMPD with sweat gland carcinoma. Previous article has reported that chemotherapy such as paclitaxel, fluorouracil, platinum, and vinblastine and molecular targeted therapy based on the genetic test results of patients have certain efficacy. Here, we report a 79-year-old male case diagnosed with human epidermal growth factor receptor 2 (HER-2) overexpression, which was effectively controlled by chemotherapy and anti–HER-2 treatment such as pyrotinib.

## 1 Introduction

The first case of Paget disease in the penis and scrotum was reported by Crocker in 1889, as well as in other parts of the body, known as extramammary Paget disease (EMPD). EMPD is a rare cutaneous intraepithelial adenocarcinoma, accounting for about 7%–10% of Paget’s disease (1). It is mostly distributed in the areas of sweat gland cells and mainly occurs in the anogenital skin of women, and male genital tract involvement was extremely rare (2, 3). The early onset of EMPD is eczema-like manifestations, which can evolve into erosive, crusted, or eczematous plaques. Clinical features of EMPD are similar to inflammatory changes, thus often delaying diagnosis and prone to misdiagnosis. EMPD is a heterogeneous tumor, often associated with other malignant tumors, 35% of patients with EMPD with accessory cancer, 27% of patients with other internal organ tumors, and 92% of EMPD tumors occurred in urogenital. Patients with EMPD usually have a good prognosis, with an expected 5-year survival rate of 60%–92% (4, 5). When EMPD invades the dermis, it can spread to local lymph nodes and distant organ (6).

Sweat gland carcinoma originates from the sweat glands, and morbidity is lower than 0.001% in all tumors (7). Clinically, the malignancy degree of sweat gland carcinoma is rather low and has slow progression; some patients have been latent for 10–20 years; sweat gland carcinoma is evenly distributed in male and female patients; the highest incidence age is 70–80 years old; most happens in the face, head and neck, axillary, chest wall, perineum, and other places, which have also been reported. The clinical presentation is mostly a painless and slow-growing solitary mass, and a few are invasive, which may induce adjacent lymph node invasion and distant metastasis. Lymph node metastasis is the most common metastatic organ, followed by lung. The 5-year survival rate is 59.38%, and the 10-year survival rate is 29.4% (8, 9).

Paget’s disease in the scrotum with sweat gland carcinoma is extremely rare. Saidi et al. reported the first known case of scrotal endocrine sweat gland carcinoma associated with extramammary gland Paget disease in1997 (10). So far, no more than 50 cases of Paget disease combined with sweat gland carcinoma at the scrotal site have been reported. The diagnosis of EMPD combined with sweat gland carcinoma mainly depends on the pathology. Paget cells with hypereratosis had abundant, transparent, and eosinophilic cytoplasm in Hematoxylin and Eosin (HE) staining. Wilkinson divided EMPD into primary type and secondary type, where EMPD can progress from non-invasive epidermal tumor to invasive adenocarcinoma, which may have the potential to spread to regional lymph nodes and distant organs, whereas secondary EMPD has associated with potential epidermal metastasis or invasion (11, 12). Cytokeratin 7 (CK7) expression was positive in the primary EMPD and negative for CK20, Human melanoma black-45 (HMB-45), and CK5/6 expression. Gross cystic disease fluid protein 15 (GCDFP-15) was positive in 30.0%–52.6% of EMPD, and its positive expression could suggest apogland-derived Paget disease (13). Sweat gland carcinoma cells express CK7, Epithelial membrane antigen (EMA), Carcinoembryonic antigen (CEA), and S-100 protein. p63 and CK5/6 are sensitive and specific for the diagnosis of sweat gland carcinoma (14). Apocrine sweat gland carcinoma and EMPD both express CK7, but there are differences between them: CK5/6 is a sensitive and specific diagnostic marker of sweat gland carcinoma, whereas EMPD combined with sweat gland carcinoma does not express CK5/6 (14, 15). Therefore, EMPD with apocrine sweat gland carcinoma is considered to originate from the apsweat gland but not pure sweat gland carcinoma.

Herein, we report an elderly male case of advanced EMPD with human epidermal growth factor receptor 2 (HER-2) overexpression combined with sweat gland carcinoma, sequentially received first-line chemotherapy and second-line anti–HER-2 Antibody-drug conjugates (ADC) drug treatment, followed by third-line treatment with third-line pan-HER family receptor tyrosine kinase inhibitor pyrotinib (Pyrotinib).

## 2 Case description

The patient was 79 years old. In May 2022, the scrotal cyst was found and then the scrotal swelling discomfort, which was initially misdiagnosed as eczema in the other hospital. When he came to our hospital, the skin of the penile scrotum was significantly thickened, covering about 4 cm × 3cm, and multiple local bulge red masses, about 1 cm × 1cm. The surface was dry, with itching, no increased skin temperature, and no skin ulceration and blisters (**Supplementary Figure 1**). Preoperative pelvic CT scan did not show significant lymph node enlargement or distant organ lesions. This patient underwent excision of a scrotal mass surgery on 24 October 2022. According to the surgical record, the described mass was located on the lower left side of the penis, measuring approximately 5 cm × 4 cm in size and in an irregular shape. Postoperative pathology report showed that squamous epithelium was large, heterogeneous cells with pale or translucent cytoplasm in scattered or nest-like distribution. The dermis showed uniform, medium-sized heterogeneous cells in solid sheets or adenoidal structures. The cytoplasm was pale, the chromatin was fine, and no pathologic nuclear schizophrenic images were seen. The combination of histomorphology and immunohistochemical labeling was consistent with poorly differentiated adenocarcinoma with Paget’s disease changes in the skin. Lesions infiltrated into the dermis and subcutaneous fat tissue. Infiltrating nerve bundles and cancerous emboli were seen in the vasculature. Intraoperative examination of the upper, lower, and right margins of the scrotal mass and postoperative examination of the margins showed carcinoma. The left margin of the scrotal mass was not found to be cancerous. Immunohistochemistry showed tumor cells expressing EMA, CK7, CEA, CD10, GCDFP-15, CerB-2 (+ +), Ki67 ~ 10% (+), approximately 10% PD-1–positive expression of lymphocytes, PD-L1 tumor cells (−), HER-2 amplification by Fluorescence in situ hybridization (FISH) (**Supplementary Figure 2**), CK5/6 (−), ER (−), and Progesterone receptor (PR) (−). Genetic testing showed that Erb-B2 receptor tyrosine kinase 2 (ERBB2) p.5310F mutation rate was 17.7%, Tumor mutational burden-high (TMB-H) was 2.87, Microsatellite instability-high (MSI-H) was 10.68%, TPS was <1%, and CPS was <1. In this case, HE-stained images clearly show the pathologic features of this patient’s Piget’s disease and sweat gland origin. Immunohistochemical staining showing double positive for CK-7 and GCDFP-15 also supports the diagnosis of EMPD combined with sweat adenocarcinoma. This patient also expressed EMA, not CK5/6, which further supports the speculation that primary EMPD originated from the gland.

One month after the surgery, the patient received PET/CT scan (**Supplementary Figure 3A**). The result showed multiple lymph node metastases in the left inguinal area, left pelvic wall, retroperitoneal aorta, and mediastinum; multiple bone metastasis in the right clavicle, sternum, multiple ribs, shoulder blades, and cervical, thoracic, and lumbar body regions; and multiple bilateral pathological rib fractures. Multiple postoperative metastases were diagnosed. Combined with the previous research and the patient’s condition, first-line treatment capecitabine (1.5 g bid) + albumin paclitaxel (400 mg) chemotherapy was started in November 2022; the evaluation of efficacy showed PR after two cycles of first-line treatment. After six cycles of chemotherapy, the patient received capecitabine maintenance. Local disease progression appeared 2 months later, with Progression-free survival (PFS) for 7 month. Based on HER-2 overexpression in this patient, second-line treatment with disitamab vedotin was started in July 2023. Disitamab vedotin was given 2 mg/kg every 3 weeks. After 2 months later, the patient developed metastatic nodules in multiple parts of the body, including scalp. No biochemical abnormalities and other adverse events such as nausea, vomiting, loss of appetite, rash, and diarrhea were observed during the administration of vedicilizumab. Evaluation of efficacy was progression disease (**Supplementary Figures 3B–D**). Then, we switched to Pyrotinib (320 mg/day).On day 2 of oral Pyrotinib administration, the patient’s skin tumors were smaller than before. After four courses of treatment, the patient’s skin tumors had significantly decreased in size and showed initial signs of re-epithelialization (**Supplementary Figures 3C, E**). Improved symptoms such as spirit, appetite, pain, and pruritus were also relieved. The adverse reaction was grade 3 diarrhea, which resolved after treatment with piretinib reduced to 240 mg/day and montmorillonite powder for diarrhea. Since the maintenance treatment with pyrrotinumab, the inguinal lymph nodes of the patient were significantly reduced, the metastatic nodules in the scalp and other places basically disappeared, and his life basically returned to normal. The performance status of ECOG score was 80. The overall patient treatment flow is shown in **Supplementary Figure 4**.

## Discussion

EMPD combined with sweat gland carcinoma is a rare disease, and extended surgical resection is the preferred treatment. However, the postoperative local recurrence rate is high, and the metastasis rate is as high as 60% (16). At present, there is a lack of standard and effective chemoradiotherapy regimen for patients with advanced EMPD combined with sweat gland carcinoma. The patient did not receive postoperative adjuvant radiotherapy because of the extensive tumor invasion, which could not be encompassed within one radiotherapy target area, as well as the patient’s old age, poor postoperative recovery, and inability to tolerate the side effects caused by radiotherapy. Previous research showed that paclitaxel, fluorouracil, platinum, and vinblastine have been used in metastatic EMPD combined with sweat gland carcinoma and have certain efficacy (17–19). In this case, the patient was treated with albumin paclitaxel + capecitabine regimen, and the best efficacy was PR, and PFS lasted for 7 months. However, after the progress, we face the difficult problem of after-line treatment choice.

Precision therapy based on molecular detection provides more choice for the treatment of EMPD. HER-2 is expressed in 61% of EMPD, and the cause of its overexpression involves amplification of ERBB2 gene on chromosome 17q21 (20). HER-2 overexpression and activated excessive signaling triggered by pathways including phosphoinositide 3 kinase/Akt/mammalian target of rapamycin (Akt/PI3K/mTOR) may lead to more malignant clinical behaviors, including lymph node metastasis, local invasion, and high recurrence rate (21, 22). In this case, the genetic test results of the patient suggested ERBB2 gene amplification, which recurred in a short term after surgery, accompanied by multiple lymph node metastases and multiple bone metastases, which showed high aggressiveness.

Recently, anti–HER-2 therapy, as an emerging anti-tumor therapy, has made breakthroughs in solid tumors such as breast cancer and gastric cancer. Anti–HER-2 agents alone or in combination with chemotherapy showed an anti-tumor effect in patients with EMPD with HER-2 overexpression. Zattarin et al. (23) summarized the efficacy of 18–single-agent trastuzumab therapy in advanced EMPD, with a median PFS up to 14 months. Trastuzumab combined with paclitaxel or vincristine for EMPD shows disease-free survival up to 36 months. There are also case reports of ADC drug ado-trastuzumab emtansine (TDM-1) in second-line treatment of advanced EMPD achieving complete response (CR) (24). In this case, the patient had poor bone marrow reserve function after first-line chemotherapy and Karnofsky Performance Status (KPS) score of 2, which was unable to tolerate traditional chemotherapy. Combined with the patient’s molecular phenotype and genetic test results, the second-line treatment of ADC drug disitamab vedotin was given. Disitamab vedotin (RC48) has the advantage of high affinity to HER-2 receptors, both direct killing and bystander effects, and cleavable linkers to target immunosuppressive cells but need more sufficient data to support it in EMPD. After two courses, the efficacy was evaluated for PD. Based on second-line anti–HER-2 ADC drug resistance, we changed the treatment regimen and administered third-line pyrotinib, a second-generation irreversible small-molecule pan-HER family receptor tyrosine kinase inhibitor targeting EGFR, HER-2, and HER-4. We have shown that pan-HER Tyrosine Kinase Inhibitor (TKI) is more capable of inhibiting the HER receptor family than conventional specific anti–HER-2 drugs. Pan-HER TKI can hinder tumor progression by targeting other receptors (e.g., EGFR) and can overcome primary resistance to some anti–HER-2 drugs (25). The phase III PHOEBE study: Pyrotinib plus capecitabine versus lapatinib plus capecitabine for the treatment of HER2-positive metastatic breast cancer (PHOEBE) confirmed that pyrotinib demonstrated good clinical efficacy in patients with progressive HER-2–positive breast cancer previously treated with trastuzumab combined with paclitaxel (26). Guo et al. applied pyrrolitinib to treat a case of scrotal EMPD harboring a triple uncommon HER-2 mutation (R678Q/S310Y/S310F) and showed a favorable efficacy (27). Our report showed that pyrotinib overcame the resistance of the anti–HER-2 ADC drug disitamab vedotin and also showed a good clinical efficacy. Based on this, anti–HER-2 therapy deserves further exploration in patients with HER-2–positive EMPD combined with sweat gland adenocarcinoma.

Regarding of the adverse effects, similar to other HER-2–targeted drugs, diarrhea was the most common adverse event of pyrotinib. Most diarrhea events were grade 1 or 2, 10.7%–15.4% were grade 3, and no grade 4 or 5 diarrhea was reported. Grade 3 diarrhea mainly occurred in the first treatment cycle, after which the incidence gradually decreased. When diarrhea occurred, montmorillonite powder or loperamide was given, and the adverse event was generally controllable (28–30). This patient developed grade 3 diarrhea during treatment with pyrrolitinib, which disappeared after reduction of pyrrolitinib to 240 mg/day and antidiarrheal treatment with montmorillonite powder. No other adverse reactions such as nausea, vomiting, skin rash, and oral mucositis were observed.

## Data availability statement

The original contributions presented in the study are included in the article/**Supplementary Material**. Further inquiries can be directed to the corresponding authors.

## Ethics statement

Written informed consent was obtained from the individual(s) for the publication of any potentially identifiable images or data included in this article.

## Author contributions

LZ: Writing – original draft, Writing – review & editing. HW: Writing – original draft, Writing – review & editing. DC: Data curation, Resources, Writing – review & editing. WW: Conceptualization, Writing – review & editing. LY: Data curation, Writing – original draft. NY: Resources, Writing – original draft. LP: Resources, Writing – original draft. PL: Resources, Writing – original draft. JZ: Writing – review & editing. BX: Writing – review & editing.
