# Supplementary material for: Case report: Pyrotinib in the treatment of advanced scrotal EMPD combined with sweat gland carcinoma
Source: Front Oncol. 2024 Jul 9;14:1382376. doi: 10.3389/fonc.2024.1382376 (PMC11263006; doi:10.3389/fonc.2024.1382376)
Supplement: Supplementary file 1 [file DataSheet_1.docx]

Supplementary Material

# Supplementary Figures and Tables

## Supplementary Figures


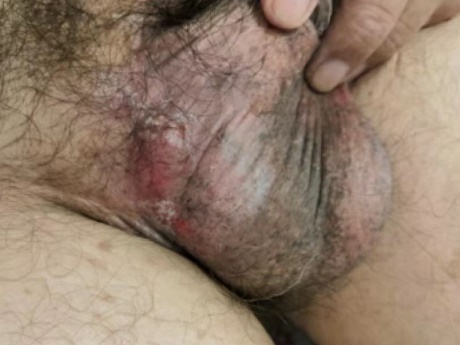


**Supplementary Figure 1.** The initial appearance of skin tumors in our patient, which was initially misdiagnosed as scrotal eczema with peeling areas.

(A)**
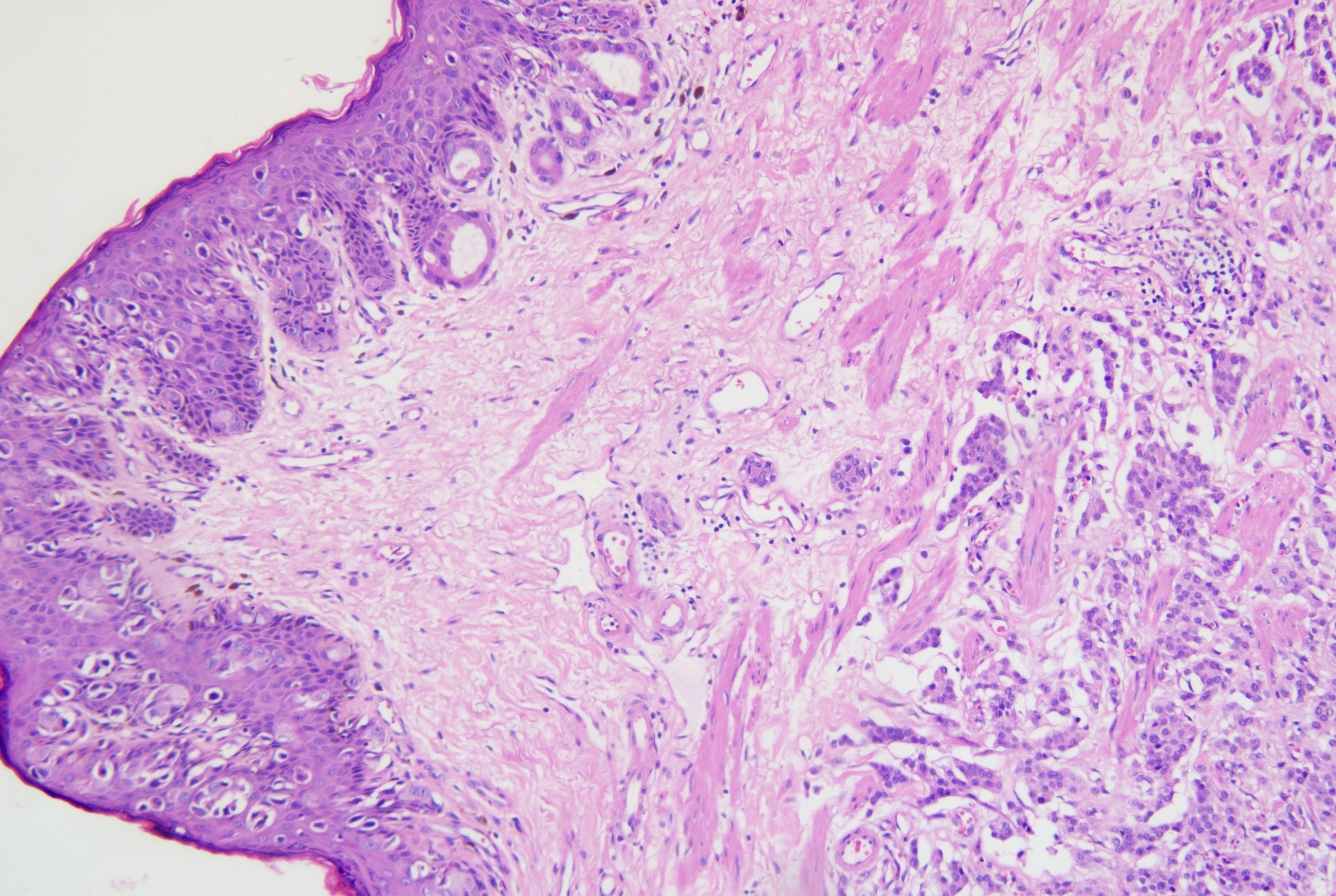
**

(B)**
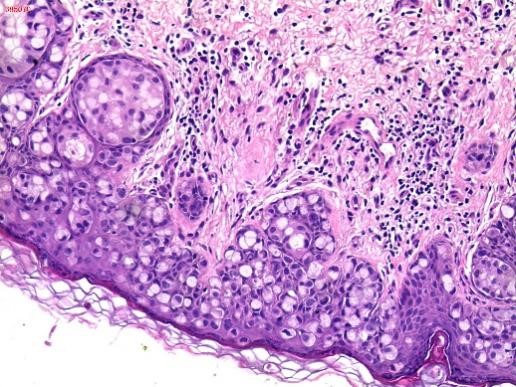
**

(C) **
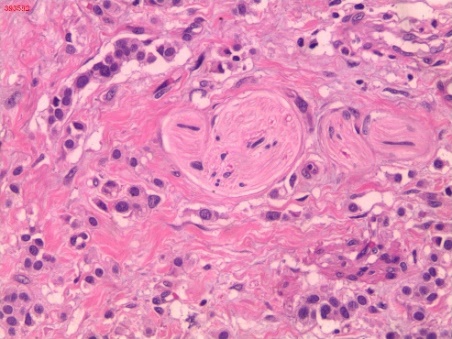
**

(D) **
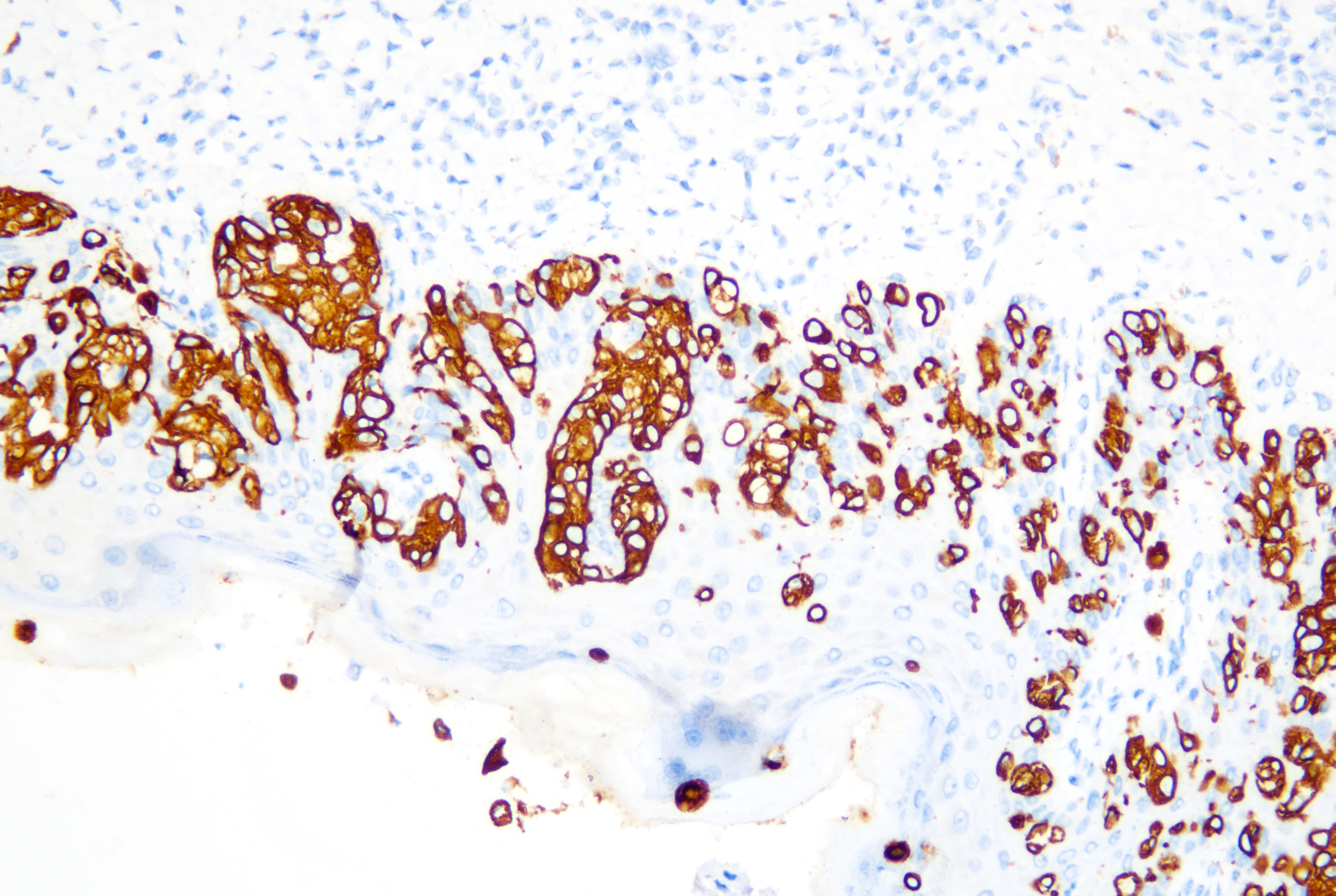
**

(E)
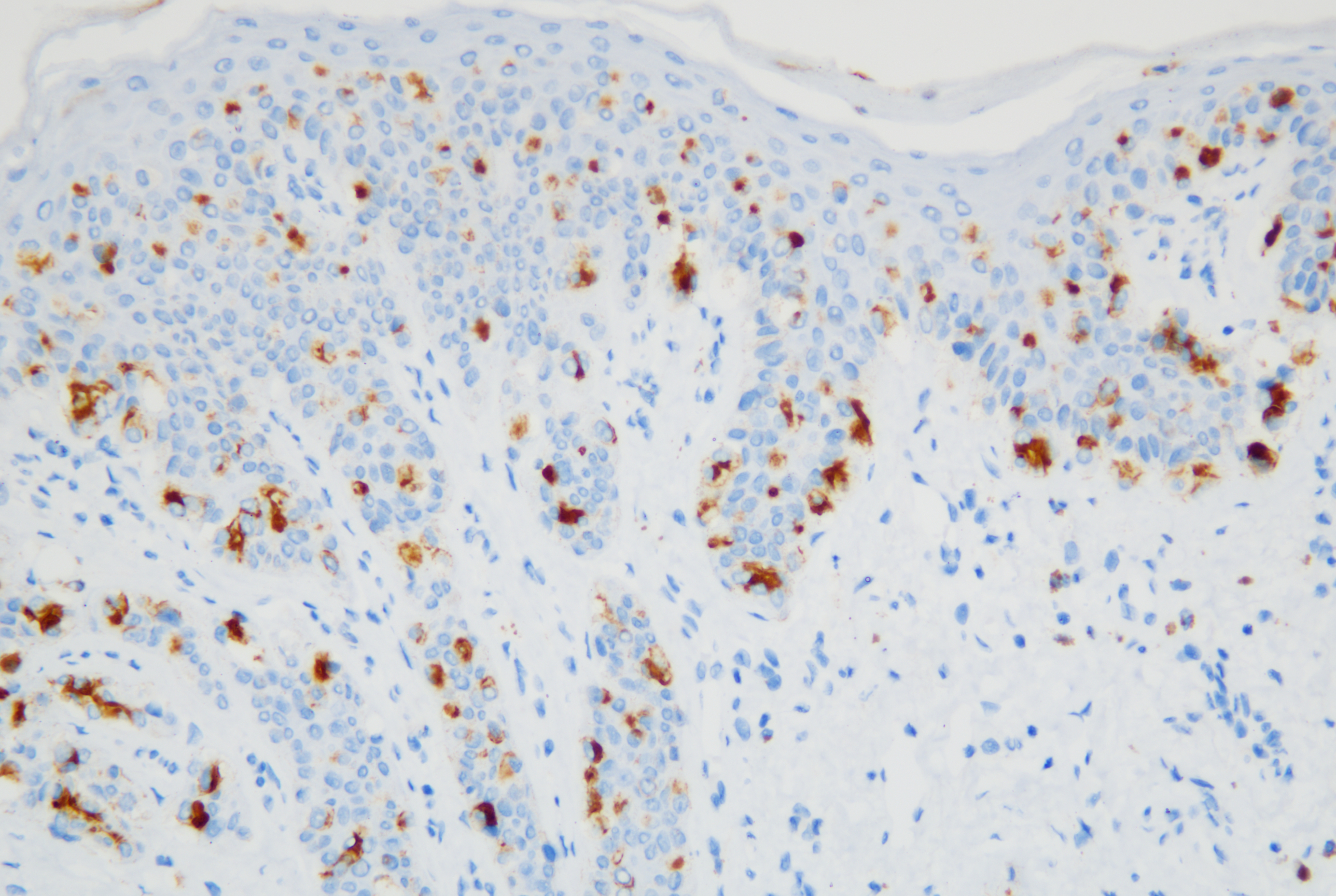


(F) **
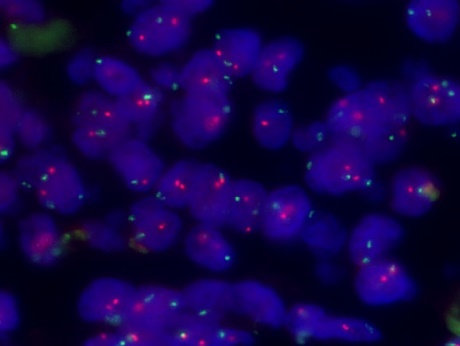
**

**Supplementary Figure 2.** (A) HE staining × 100.(B) HE staining × 200. (C) HE staining × 400. (D) Immunohistochemical membrane antigen EMA (+), cytokeratin CK7 (+) .(E) Immunohistochemical GCDFP-15 (+).(F)FISH detection: HER-2 amplification.

(A)
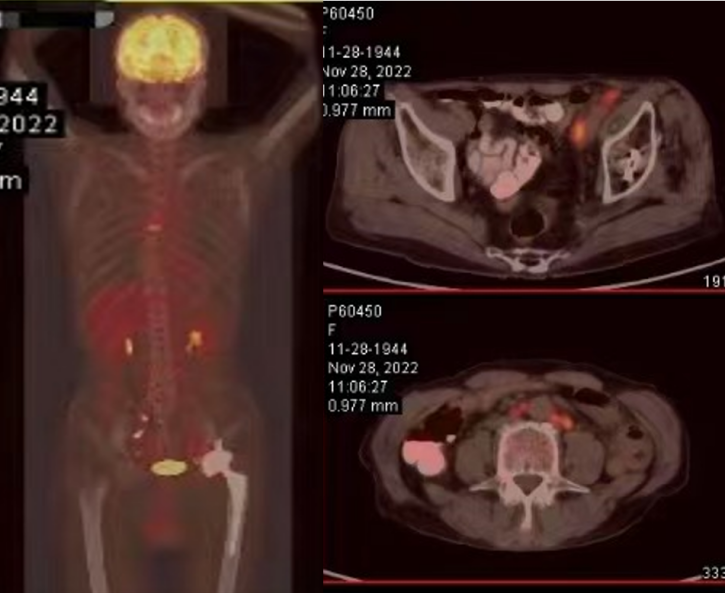


(B)
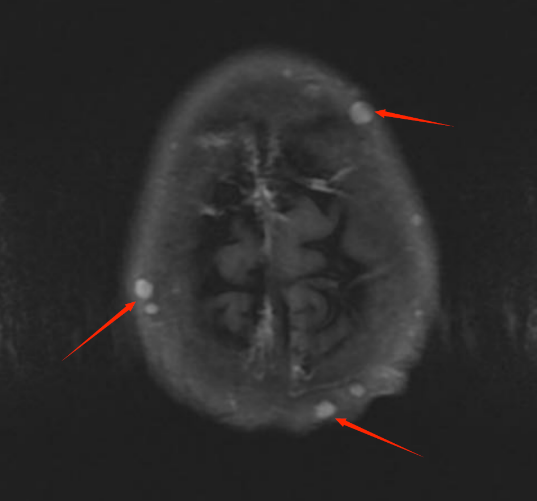


(C)
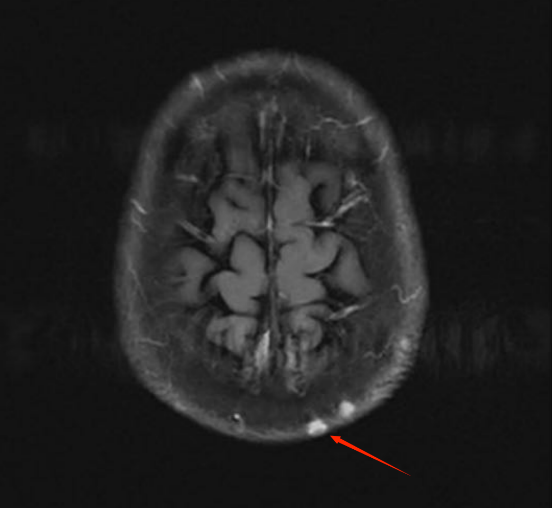


(D)
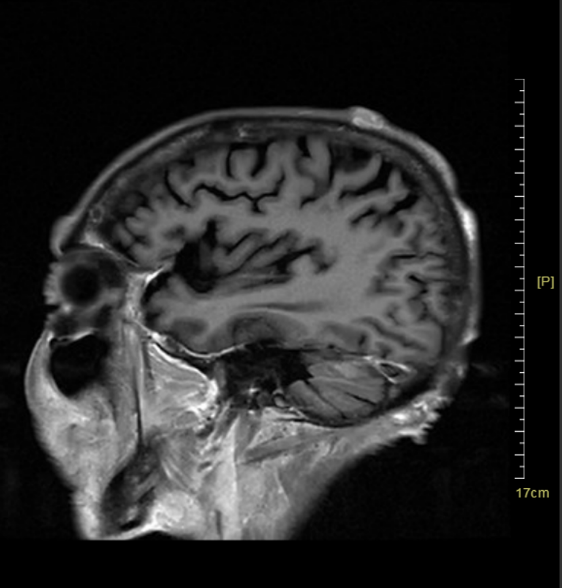


(E)
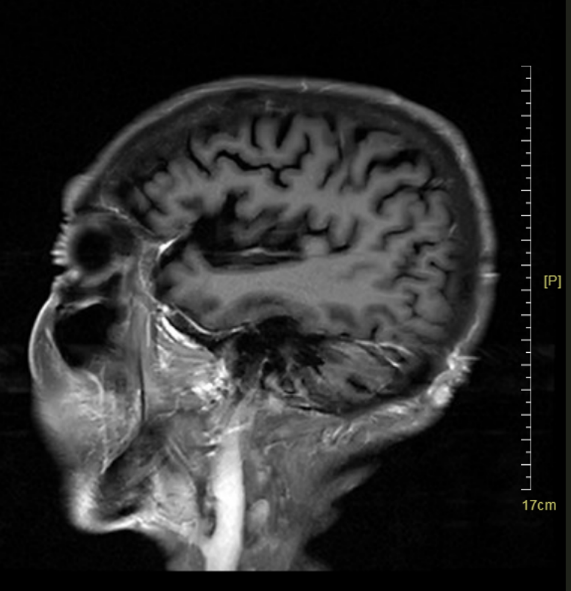


**Supplementary Figure 3.** The imaging findings of Skin Tumors at Different Phases of Pyrotinib Therapy: (A)FDG PET/CT scan at baseline of patient’s clinical history. (B.D) Two courses of treatment with Disitamab Vedotin As the disease progresses, multiple scattered skin masses appear throughout the body, with particularly severe on the scalp. (C.E) After 4 courses of Pyrotinib maintenance therapy, the skin tumors decreased in size and gradually showed an epithelial trend.


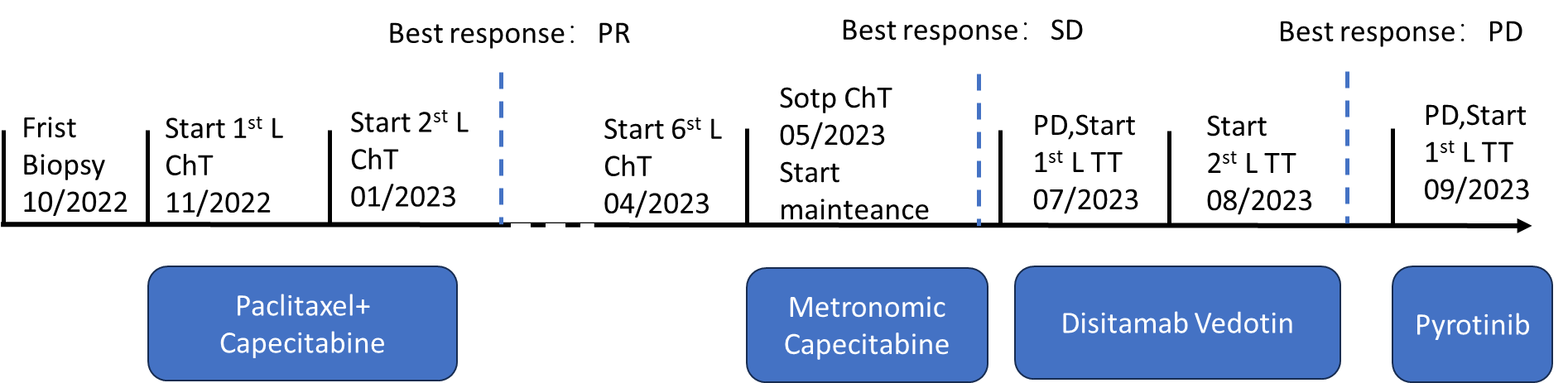


**Supplementary Figure 4.** Timeline of systemic treatments administrated during patient’s disease history. ChT, chemotherapy; TT, Targted therapy; PD, progression disease; PR, partial disease response.
